# Quantitative Assessment of Common Genetic Variants on *FOXE1* and Differentiated Thyroid Cancer Risk

**DOI:** 10.1371/journal.pone.0087332

**Published:** 2014-01-29

**Authors:** Hongling Zhu, Qian Xi, Lianyong Liu, Jingnan Wang, Mingjun Gu

**Affiliations:** Department of Endocrine, Shanghai Pudong New Area Gongli Hospital, Shanghai, People's Republic of China; Institut Jacques Monod, France

## Abstract

Forkhead box E1 encodes the transcription factor FOXE1 (or TTF-2), which together with Homeobox protein NKX2-1, PAX8 and HHEX, are pivotal proteins required for thyroid gland formation, differentiation and function. Recently, genome-wide association studies have identified *FOXE1* as a thyroid cancer (TC) susceptibility gene in populations of European descent. After that, a number of studies reported that the rs965513, rs1867277, and rs71369530 polymorphism in *FOXE1* has been implicated in TC risk. However, the causal variants remain unknown. To derive a more precise estimation of the relationship, a meta-analysis of 9,828 TC cases and 109,995 controls from 14 case–control studies was performed. Overall, significant results were observed for rs965513 (OR = 1.71, 95% CI: 1.59–1.85, P<10^−5^), rs1867277 (OR = 1.64, 95% CI: 1.51–1.78, P<10^−5^) and rs71369530 (OR = 2.01, 95% CI: 1.66–2.44, P<10^−5^) polymorphism. In the subgroup analysis by ethnicity, we found that rs965513 polymorphism confer high risk for Caucasians with per-allele OR of 1.80 (95% CI: 1.69–1.92, P<10^−5^) compared to East Asians of 1.35 (95% CI: 1.09–1.67, P = 0.006). There was strong evidence of heterogeneity, which largely disappeared after stratification by ethnicity. In the subgroup analysis by sample size, and study design, significantly increased risks were found for the polymorphism. In conclusion, this meta-analysis demonstrated that common variations of *FOXE1* are a risk factor associated with increased TC susceptibility.

## Introduction

Thyroid cancer (TC) is the most common endocrine malignancy, and accounts for 1% of all neoplasias [1]. TC is classified histologically into four main groups: papillary (PTC), follicular (FTC), medullary (MTC) and undifferentiated thyroid carcinomas. Most of all thyroid tumors are PTC (80–85%) or FTC (10–15%) [2]. Although the etiology of this cancer is not well characterized, thyroid cancer is believed to be a complex disease, in which common genetic variants located in low penetrance genes may interact with each other and with the environment, determining individual susceptibility. Among the latter, ionizing radiation, especially exposure to fallout of radioactive iodine isotopes in childhood, strongly predisposes to TC [3]. The contribution of genetics to the risk of thyroid cancer is greater than to any other cancer, and the effect extends beyond the nuclear family [4], [5]. The identification and further assessment of the relevant genetic variations are important for understanding the potential mechanisms involved in thyroid carcinogenesis.

Recently, spectacular advance was made in identifying susceptible genes involved in TC through genome-wide association strategy (GWAS) among European descent [6], [7]. A number of recent studies have identified single nucleotide polymorphisms (SNPs) associated with TC risk on chromosomes 5q24, 8q24, 9q22, and 14q13 [6], [8]–[10]. Common genetic variant (rs965513) of *FOXE1* at chromosome 9q22, has been identified as a new hotspot for thyroid cancer susceptibility by a recent GWA study [6], [7]. *FOXE1* possesses a polymorphic polyalanine tract (rs71369530) just distal to its DNA-binding domain, with 11–22 alanine residues reported, although *FOXE1* 14Ala and *FOXE1* 16Ala account for greater than 98% of reported alleles [11]. Recently, Landa et al. [12] found strong evidence that one SNP located in the promoter region of the *FOXE1* gene (rs1867277) was positively associated with sporadic thyroid cancer susceptibility. Over the past few years, these polymorphisms (rs965513, rs1867277, and rs71369530) in the *FOXE1* region and thyroid cancer risk have been independently replicated by subsequent studies. As stated by McClellan and King, many if not most of the genetic polymorphisms that are reported to be associated with common disorders in GWA studies are factually spurious associations caused by subtle differences in ancestry between the populations being studied (population stratification) [13]. The interpretation of these studies has been further complicated by the use of different ethnic populations, insufficient power, small effect of the polymorphism on thyroid cancer risk and phenotypic heterogeneity. In addition, with the increased studies in recent years among East Asian populations, there is a need to reconcile these data. We therefore performed a meta-analysis of the published studies to clarify this inconsistency and to establish a comprehensive picture of the relationship between common variants on chromosome *FOXE1* and thyroid cancer.

## Materials and Methods

### Literature search strategy and selection criteria

Epidemiological genetic association studies published before the end of Nov. 2013 on thyroid cancer and polymorphism in the *FOXE1* gene were sought by computer-based searches from databases including Pubmed, SCOPUS, ISI web of knowledge, Embase, Cochrane databases and CNKI (China National Knowledge Infrastructure) without language restriction. Search term combinations were keywords relating to the *FOXE1* gene (e.g., “*FOXE1*”, “*TTF-2*”, “9q22”, “rs965513”, “rs1867277”, “rs71369530”, “polyalanine tracts”) in combination with words related to thyroid cancer (e.g., “thyroid cancer”, “thyroid carcinoma”, “thyroid tumor”). We replaced one term each time until all possible combination mode were searched to avoid any missing literature. The titles and abstracts of potential articles were screened to determine their relevance, and any clearly irrelevant studies were excluded. The full texts of the remaining articles were read to determine whether they contained information on the topic of interest. Furthermore, reference lists of primary studies and review articles were also reviewed by a manual search to identify additional relevant publications.

The included studies have to meet the following criteria: (1) evaluation of at least one of those three polymorphisms (rs965513, rs1867277, rs71369530) and thyroid cancer risks using case–control or cohort design, (2) original papers containing independent data, (3) identification of thyroid cancer patients was confirmed histologically or pathologically, (4) genotype distribution information or odds ratio (OR) with its 95% confidence interval (CI) and P-value, and (5) genotype distribution of control group must be consistent with Hardy–Weinberg equilibrium (HWE). The major reasons for exclusion of studies were (1) overlapping data, (2) case-only studies, and (3) review articles.

### Data extraction

Information was carefully extracted from all eligible publications independently by the two authors according to the inclusion criteria listed above. For each included study, the following information was extracted from each report according to a fixed protocol: first author, publication year, definition and numbers of cases and controls, frequency of genotypes, age, sex, ethnicity, HWE status, source of control, histological subtypes and genotyping method. Review reports from the two were then compared to identify any inconsistency, and differences were resolved by further discussion among all authors. Studies with different ethnic groups were considered as individual studies for our analyses. Meanwhile, different case–control groups in one study were considered as independent studies. The instrument “Extended-quality score” was used to assess the quality of association studies [14]. In general, each article is scored on an extended-quality scale that designates them as “high,” “median,” or “poor” quality.

### Statistical methods

Crude ORs with the corresponding 95% CIs were used to assess the strength of the association between the *FOXE1* polymorphism and thyroid cancer risks. The per-allele OR of the risk allele was estimated. Then, we estimated the risks of the heterozygous and homozygous genotypes on TC compared with the wild-type homozygote [15]. Cochran's χ^2^ based Q-statistic and I^2^ test [16], [17] test was performed to assess possible heterogeneity in the combined studies. Generally, I^2^ values <25% correspond to no or little heterogeneity, values 25–50% correspond to moderate heterogeneity, and values >50% correspond to strong heterogeneity between studies. Random-effects and fixed-effect summary measures were calculated as inverse-variance-weighted average of the log odds ratio [18]. The results of random-effects summary were reported in the text because it takes into account the variation between studies. 95% CIs were constructed using Woolf's method [19]. Sources of heterogeneity were investigated by stratified meta-analyses based on ethnicity, sample size (No. cases ≥500 or, <500) and study design strategy (GWAS vs. candidate gene). Ethnic group was defined as East Asians (i.e., Chinese, Japanese, and Korean), and Caucasians (i.e. people of European origin). In addition, ethnicity, sample size, and study design (GWAS vs. candidate gene) was analyzed as covariates in meta-regression. The significance of the pooled OR was determined by Z test. Publication bias was assessed with the Egger test and Begg test [20], [21]. Sensitivity analysis was performed by removing each individual study in turn from the total and re-analyzing the remainder. The analysis was conducted using the STATA software version 10.0 (Stata Corporation, College Station, TX). All the P-values were for two-sided analysis and values of P<0.05 were considered statistically significant.

## Results

### Study Characteristics

The combined search yielded 96 references. Figure S1 shows the study selection process. Finally, a total of 14 eligible association studies with 9,828 TC cases and 109,995 controls were identified [6], [7], [11], [12], [22]–[31], with 4 studies genotyping more than one variant. There are 19 data sets from 12 studies with 8,602 cases and 102,846 controls concerning rs965513, and 7 data sets from 5 studies involving 2,017 cases and 13,281 controls concerning rs1867277. For the rs71369530 polymorphism, 5 data sets from 4 studies involved a total of 448 cases and 746 controls. Of the cases, 77% were Caucasian, and 23% were East Asian. Eleven studies were given high quality, and three studies were given median quality. No ‘poor quality’ study was found. The detailed characteristics of the studies included in this meta-analysis are shown in Table 1 (Figure S2).

**Table 1:** Characteristics of the studies included in the meta-analysis.

Study	Year	Polymorphism	Ethnicity	Cases	Controls	No. of cases/controls	Genotyping method	Quality
Gudmundsson [6]	2009	rs965513	European, American	Histologically confirmed thyroid cancer	Cancer-free individuals	962/38923	SNP arrays, SNaPshot	High
Kallel [11]	2010	PolyAla	Spanish	Pathologically confirmed papillary thyroid cancer	General population	170/218	Sequencing	Median
Landa [12]	2009	rs1867277	Spanish, Italian	Pathologically confirmed papillary thyroid carcinoma	General population	984/1028	GoldenGate	High
Takahashi [22]	2010	rs965513	Belarusian, Russian	Histologically confirmed papillary thyroid carcinoma	General population	660/1268	SNP arrays, TaqMan	High
Matsuse [23]	2011	rs965513	Japanese	Histologically confirmed papillary thyroid carcinoma	General population	479/2764	TaqMan	High
Denny [24]	2011	rs965513	American	Pathologically confirmed thyroid cancer	Cancer-free individuals	96/6274	SNP arrays	High
Gudmundsson [7]	2012	rs965513	European, American	Histologically confirmed thyroid cancer	Cancer-free individuals	558/43108	SNP arrays, SNaPshot	High
Tomaz [25]	2012	rs965513, rs1867277, PolyAla	Portuguese	Pathologically confirmed non-medullary thyroid cancer	General population	140/130	Sequencing	Median
Bullock [26]	2012	rs1867277, PolyAla	Australian, British	Pathologically confirmed papillary thyroid cancer	General population	70/5733	Sequencing, TaqMan	High
Jones [27]	2012	rs965513, rs1867277	British	Histologically confirmed non-medullary thyroid cancer	General population	753/6120	Kaspar	High
Wang [28]	2013	rs965513	Chinese	Histologically confirmed papillary thyroid cancer	Cancer-free individuals	1348/1005	SNaPshot	High
Liyanarachchi [29]	2013	rs965513	Polish, American	Histologically confirmed papillary thyroid carcinoma	General population	2494/2264	SNaPshot, iPLEX	High
Köhler [30]	2013	rs965513	Italian	Histologically confirmed papillary thyroid cancer	General population	690/497	SNP arrays	High
Damiola [31]	2013	rs965513, rs1867277, PolyAla	Byelorussian	Histologically confirmed papillary thyroid carcinoma	General population	70/303	HRM, TaqMan, PCR	High
Unpublished data	/	rs965513	Chinese	Histologically confirmed papillary thyroid carcinoma	General population	354/360	iPLEX	Median

### Association of rs965513 polymorphism with thyroid cancer

**Figure 1:**
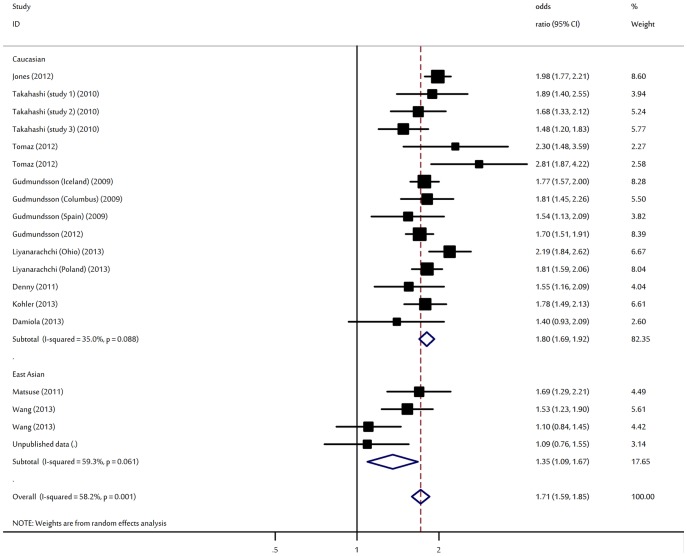
Forest plot for association of *FOXE1* rs965513 polymorphism and thyroid cancer risk.

There was a wide variation in the A allele frequency of the rs965513 polymorphism among the controls across different ethnicities, ranging from 0.06 to 0.44 (Figure S2). For East Asian controls, the A allele frequency was 0.06 (95% CI: 0.05–0.08), which was lower than that in Caucasian controls (0.39; 95% CI: 0.34–0.44). For TC risk and the rs965513 polymorphism, our meta-analysis gave an overall OR of 1.71 (95% CI: 1.59–1.85, P<10^−5^; Figure 1) with statistically significant between-study heterogeneity (P = 0.001). Significantly increased TC risks were also found for those heterozygous (OR = 2.17, 95% CI: 1.86–2.57; P<10^−5^) and homozygous for the risk A allele (OR = 2.95, 95%CI: 2.29–3.94; P<10^−5^) when compared with the wild type genotype.

In view of significant heterogeneity and to seek for its potential sources, we performed a panel of subgroup analyses on ethnicity and sample size. When studies were stratified for ethnicity, significant risks were found among Caucasians in all comparisons (A allele: OR = 1.80, 95% CI: 1.69–1.92, P<10^−5^; heterozygous: OR = 2.60, 95% CI: 2.06–3.02, P<10^−5^; homozygote: OR = 3.36, 95% CI: 2.75–4.51, P<10^−5^). Similar significant associations were also observed for East Asians with per-allele OR of 1.35 (95% CI: 1.09–1.67, P = 0.006). Analysis restricted to the 8 studies with at least 500 cases, which should be less prone to selective publication than smaller studies, yielded an OR of 1.75 (95% CI: 1.59–1.93, P<10^−5^). As for smaller studies, the summary OR of rs965513-A variant for TC was 1.67 (95% CI: 1.48–1.88, P<10^−5^). By considering study design subgroups, the OR was 1.75 (95% CI: 1.61–1.90, P<10^−5^) in GWAS compared to 1.70 (95% CI: 1.53–1.88, P<10^−5^) in candidate gene studies (Table 2). When subgroup analyses by histological types were considered (6, 833 cases and 47, 384 controls from 8 studies), the polymorphism had a significantly increased risk for papillary thyroid cancer with an OR of 1.65 (95% CI: 1.50–1.81, P<10^−5^; I^2^ = 59%, P _heterogeneity_ = 0.003).

**Table 2:** Main results of overall and subgroups in the meta-analysis.

Polymorphism (risk allele)	Total/Subgroup analysis	No. of data sets	No. of cases/controls	Risk allele					Heterozygous					Homozygous				
				OR (95%CI)	P(Z)	P(Q)^a^	I^2^ (%)	P(Q)^b^	OR (95%CI)	P(Z)	P(Q)^a^	I^2^ (%)	P(Q)^b^	OR (95%CI)	P(Z)	P(Q)^a^	I^2^ (%)	P(Q)^b^
rs965513 (A)	Total	19	8602/102846	1.71 (1.59–1.85)	<10^−5^	0.001	58		2.17 (1.86–2.57)	<10^−5^	<10^−4^	67		2.95 (2.29–3.94)	<10^−5^	<10^−4^	77	
	Ethnicity							<10^−4^					<10^−4^					<10^−4^
	Caucasian	15	6521/98717	1.80 (1.69–1.92)	<10^−5^	0.09	35		2.60 (2.06–3.02)	<10^−5^	0.003	47		3.36 (2.75–4.51)	<10^−5^	0.0005	61	
	East Asian	4	2181/4129	1.35 (1.09–1.67)	0.006	0.06	59		1.68 (1.24–2.12)	<10^−4^	0.01	38		1.95 (1.51–3.21)	<10^−4^	0.003	55	
	Sample size							0.09					0.05					0.05
	No. cases ≥500	8	6420/90190	1.75 (1.59–1.93)	<10^−5^	0.002	70		2.27 (1.93–2.72)	<10^−5^	0.007	43		3.16 (2.59–4.28)	<10^−5^	<10^−4^	79	
	No. cases < 500	11	2182/12656	1.67 (1.48–1.88)	<10^−5^	0.06	43		1.97 (1.59–2.41)	<10^−5^	0.004	68		2.76 (2.00–3.65)	<10^−5^	0.001	75	
	Design strategy							0.83					0.65					0.51
	GWAS	5	1766/44587	1.75 (1.61–1.90)	<10^−5^	0.89	0		2.35 (1.97–2.84)	<10^−5^	0.77	0		3.03 (2.52–3.92)	<10^−5^	0.51	0	
	Candidate gene	14	6836/58259	1.70 (1.53–1.88)	<10^−5^	<10^−4^	69		2.00 (1.68–2.30)	<10^−5^	<10^−4^	63		2.88 (2.21–4.07)	<10^−5^	<10^−4^	78	
rs1867277 (A)	Total (All Caucasian)	7	2017/13281	1.64 (1.51–1.78)	<10^−5^	0.39	4		1.86 (1.57–2.23)	<10^−5^	0.26	9		2.63 (1.98–3.51)	<10^−5^	0.08	19	
Poly-Ala (16-Ala)	Total (All Caucasian)	5	448/746	2.01 (1.66–2.44)	<10^−5^	0.29	20		/	/	/			/	/	/		

a Cochran's chi-square Q statistic test used to assess the heterogeneity in subgroups.

b Cochran's chi-square Q statistic test used to assess the heterogeneity between subgroups.

Significant heterogeneity was present among the 19 data sets (P<0.05). In meta-regression analysis, sample size (P = 0.68), and study design (P = 0.89), did not significantly explained such heterogeneity. By contrast, ethnicity (P = 0.001) was significantly correlated with the magnitude of the genetic effect.

### Association of rs1867277 polymorphism with thyroid cancer

The A allele frequency in Caucasians was 0.40 (95% CI: 0.38–0.43). Using random effect model, the per-allele overall OR of the A variant for TC was 1.64 (95% CI: 1.51–1.78, P<10^−5^; Figure S3), with corresponding results for heterozygous and homozygous of 1.86 (95% CI: 1.57–2.23, P<10^−5^) and 2.63 (95% CI: 1.98–3.51, P<10^−5^), respectively.

### Association of polyAla (rs71369530) polymorphism with thyroid cancer

The two most common alleles were the 14-alanine and the 16-alanine alleles, occurring with a frequency of 58.5% and 32.3% in Caucasian controls, respectively. Among the polyAla (rs71369530) alleles, 16-Ala was the alleles showing higher frequencies in cases than controls. The overall per-allele OR of the 16-Ala variant for total TC was 2.01 (95% CI: 1.66–2.44, P<10^−5^; Figure S4),

### Sensitivity analyses and publication bias

Sensitivity analysis indicated that no single study influenced the pooled OR qualitatively, suggesting that the results of this meta-analysis are stable (Figure S5–S7). The shape of the funnel plots was symmetrical for these polymorphisms (Figure S8–S10). The statistical results still did not show publication bias in these studies for rs965513 (Egger's test, P = 0.21), rs1867277 (Egger's test, P = 0.92) and rs71369530 polymorphism (Egger's test, P = 0.19).

## Discussion

GWAS have led to the identification of multiple new genetic variants associated with thyroid cancer risk. Most of these thyroid cancer GWAS and replication studies have been conducted in European populations [6], [7], [25]–[27], [30] and to a lesser extent in East Asians [23], [28]. Replication of initial GWAS findings is considered a gold standard for reporting genotype–phenotype associations. Besides, there are significant differences in allele frequencies and the prevalence of thyroid cancer among different ethnic populations. It is, therefore, important to quantitatively assess the effects of the GWAS-identified markers in different ethnic populations and to explore potential heterogeneity of published data. To the best of our knowledge, this is the first comprehensive meta-analysis examining the common variations on FOXE1 gene and its relationship to susceptibility for thyroid cancer. Its strength was based on the accumulation of published data giving great information to detect significant differences. In total, the meta-analysis involved 14 studies which provided 9,828 TC cases and 109,995 controls. Our results demonstrated that the 3 polymorphisms (rs965513, rs1867277, rs71369530) of *FOXE1* is a risk factor for developing TC.

Genetic heterogeneity is inevitable in disease identification strategy [32]. As for rs965513 polymorphism, we identified ethnicity as a potential source of between-study heterogeneity by subgroup analysis and meta-regression. In the stratified analysis by ethnicity, we observed that association between rs965513 polymorphism and risk for TC in Caucasians (OR = 1.80) was stronger than that in East Asian populations (OR = 1.35). Here are several explanations to interpret above-mentioned phenomenon. Firstly, ethnic differences may attribute to these different results, since the C allele distributions of the rs965513 polymorphism varies between Caucasians, and East Asians, with a prevalence of ∼37%, and ∼11%, respectively [6], [28]. On the other hand, different populations usually have different linkage disequilibrium patterns. A polymorphism may be in close linkage with another nearby causal variant in one ethnic population but not in another [33]. Moreover, it is possible that variation at this locus has modest effects on TC, but environmental factors may predominate in its progress, and mask the effects of this variation. Specific environmental factors like ionizing radiation and deficiency in iodine intake that have been already well studied in recent decades [34]. Most of included studies did not consider those important environmental factors. It is still unknown whether the lifestyle characteristics of different populations influence the association between the polymorphisms and TC. The unconsidered factors mixed together may cover the role of the polymorphism in East Asian populations.

FOXE1 is important for both pituitary- and thyroid- gland formation [35], [36] and is at the center of a regulatory network of transcription factors and cofactors that initiate thyroid differentiation at the embryonic stage [37]. Furthermore, mutations of the *FOXE1* gene cause human syndromes that are associated with thyroid agenesis, among other phenotypes [38]. FOXE1 is also necessary for the maintenance of the differentiated state of the thyroid, as it is involved in regulating the transcription of thyroid-specific genes, such as the thyroglobulin and thyroperoxidase genes. Furthermore, the expression of *FOXE1* has been shown to be abnormal in thyroid tumors [39]. The 9q22.33 SNP rs965513 was first reported in a GWAS of TC in a European population, and has since been replicated in several later studies [6], [7], [28]. It has been suggested to tag a functional variation near the *FOXE1* gene which contributes to an increased risk of developing thyroid cancer. Besides, the variant has also been associated with low serum concentrations of thyroid-stimulating hormone, and free thyroxin [7].

A significant association with TC was also found for the *FOXE1* 16-Ala and rs1867277 variant in the present meta-analysis. Carré et al. reported that *FOXE1* with 16-Ala induced a stronger transactivation of the thyroglobulin promoter than the 14-Ala variant [40]. These results suggest a functional consequence for the presence of polyAla expansions (>14), but not for contractions (≤14). However, a recent study reported a modest transcriptional impairment of 16-Ala *FOXE1*, when compared with the function of the 14-Ala variant, on *FOXE1* responsive promoters, which was not attributable to differences in DNA binding [26]. In the case of rs1867277, the sequence with the A allele was shown to increase the transcriptional activity of the *FOXE1* gene promoter, by recruitment of leucine zipper upstream stimulatory factors 1 and 2 [12]. Thus, it is hypothesized that up-regulation of *FOXE1* could have a role in the malignant behavior of thyroid cells.

In interpreting the results, some limitations of this meta-analysis should be addressed. Firstly, our results were based on unadjusted estimates, while a more precise analysis should be conducted if all individual raw data were available, which would allow for the adjustment by other co-variants including age, sex, cigarette consumption, and other lifestyle. Secondly, the vast majority of subjects in the study are of European descent, and statistical power for analyses in other ethnicities is limited. Because the sample size was considerably smaller for East Asians studies, the main conclusions from this manuscript are based on analyses among Caucasian populations. Future studies including larger numbers of East Asians or Africans are necessary to clarify the consistency of findings across ethnic groups. Thirdly, meta-analysis is a type of retrospective study, and the recall and selection bias might exist.

Despite these limitations, this meta-analysis suggests that the three common variations on *FOXE1* (rs965513, rs1867277, rs71369530) was significantly associated with increased risk of TC, particularly in Caucasian population. As studies among other ethnic populations are currently limited, further studies including a wider spectrum of subjects to investigate the role of those variants in other populations will be needed. Besides, future studies are recommended to identify the possible gene-gene and gene-environmental interactions in this association.

## Supporting Information

Figure S1 Study selection process.
(TIF)

Figure S2Frequencies of the risk alleles of *FOXE1* rs965513 among controls stratified by ethnicity. The “
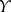
” represent outlier.(TIF)

Figure S3 Forest plot for association of *FOXE1* rs1867277 polymorphism and thyroid cancer risk.
(TIF)

Figure S4 Forest plot for association of *FOXE1* polyAla variant (71369530) and thyroid cancer risk.
(TIF)

Figure S5 Result of sensitivity analyses for *FOXE1* rs965513 polymorphism and thyroid cancer risk.
(TIF)

Figure S6 Result of sensitivity analyses for *FOXE1* rs1867277 polymorphism and thyroid cancer risk.
(TIF)

Figure S7 Result of sensitivity analyses for *FOXE1* rs71369530 polymorphism and thyroid cancer risk.
(TIF)

Figure S8 Begg's funnel plot for publication bias in studies on *FOXE1* rs965513 polymorphism and thyroid cancer.
(TIF)

Figure S9 Begg's funnel plot for publication bias in studies on *FOXE1* rs1867277 polymorphism and thyroid cancer.
(TIF)

Figure S10 Begg's funnel plot for publication bias in studies on *FOXE1* rs71369530 polymorphism and thyroid cancer.
(TIF)

Checklist S1 (DOC)
