# Supplementary figures and images for: Quantitative Assessment of Common Genetic Variants on FOXE1 and Differentiated Thyroid Cancer Risk
Source: PLoS One. 2014 Jan 29;9(1):e87332. doi: 10.1371/journal.pone.0087332 (PMC3906140; doi:10.1371/journal.pone.0087332)

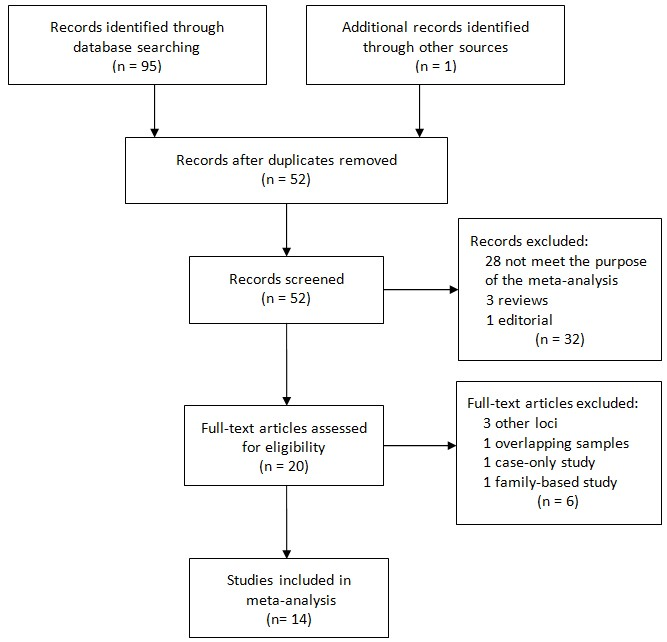

Supplement: Figure S1 — Study selection process. (TIF) [file pone.0087332.s001.tif]

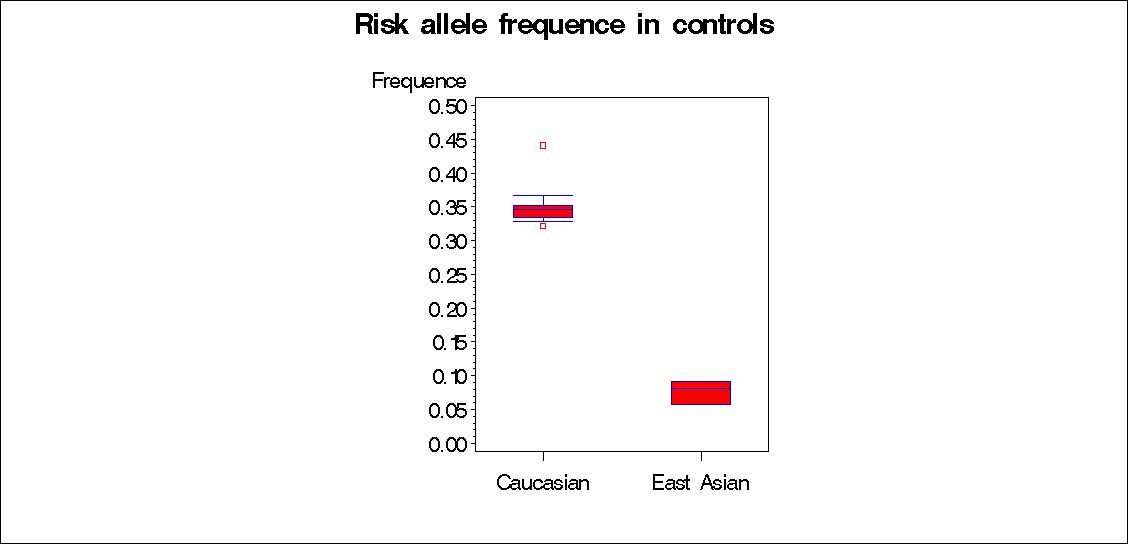

Supplement: Figure S2 — Frequencies of the risk alleles of FOXE1 rs965513 among controls stratified by ethnicity. The “” represent outlier. (TIF) [file pone.0087332.s002.tif]

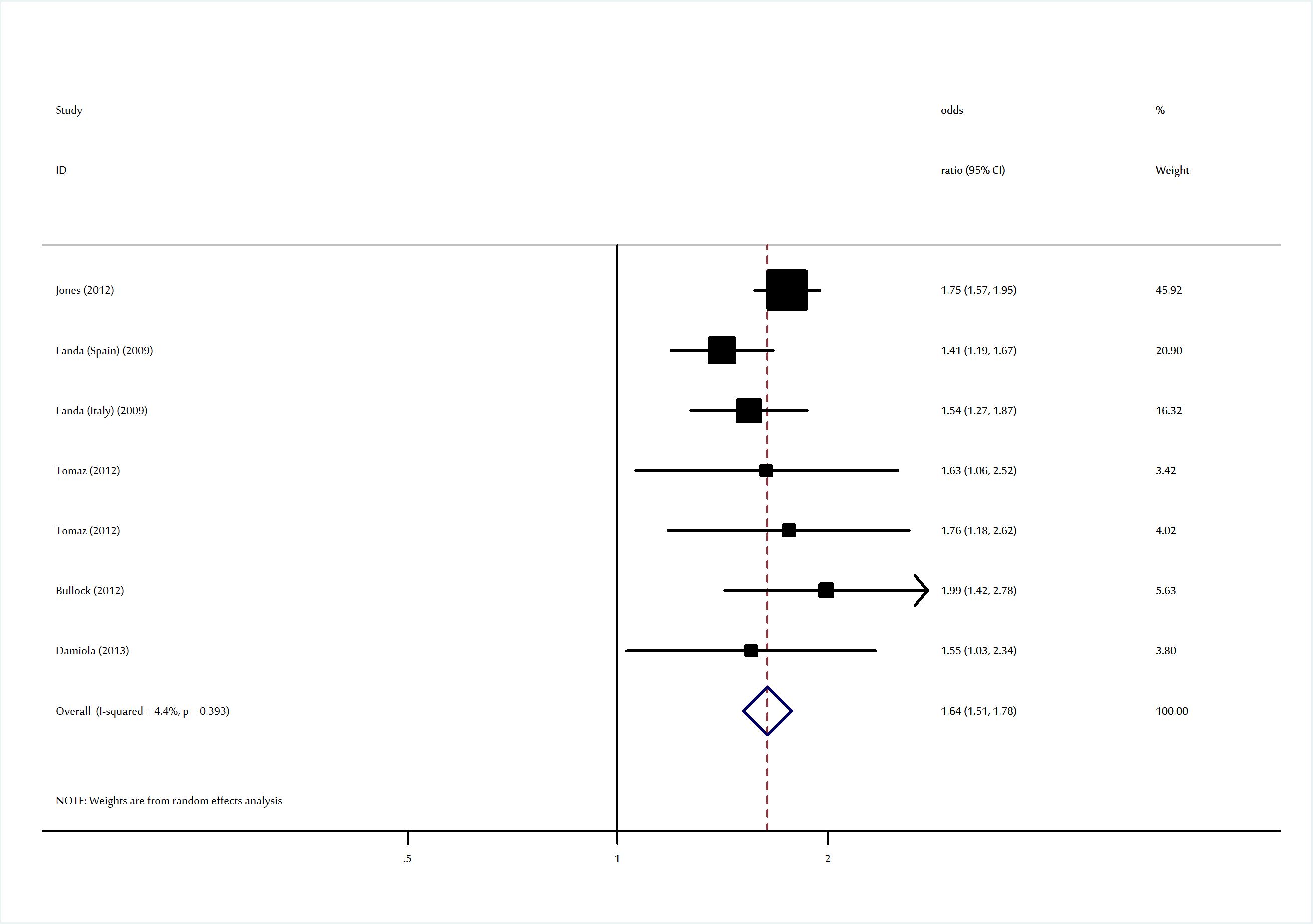

Supplement: Figure S3 — Forest plot for association of FOXE1 rs1867277 polymorphism and thyroid cancer risk. (TIF) [file pone.0087332.s003.tif]

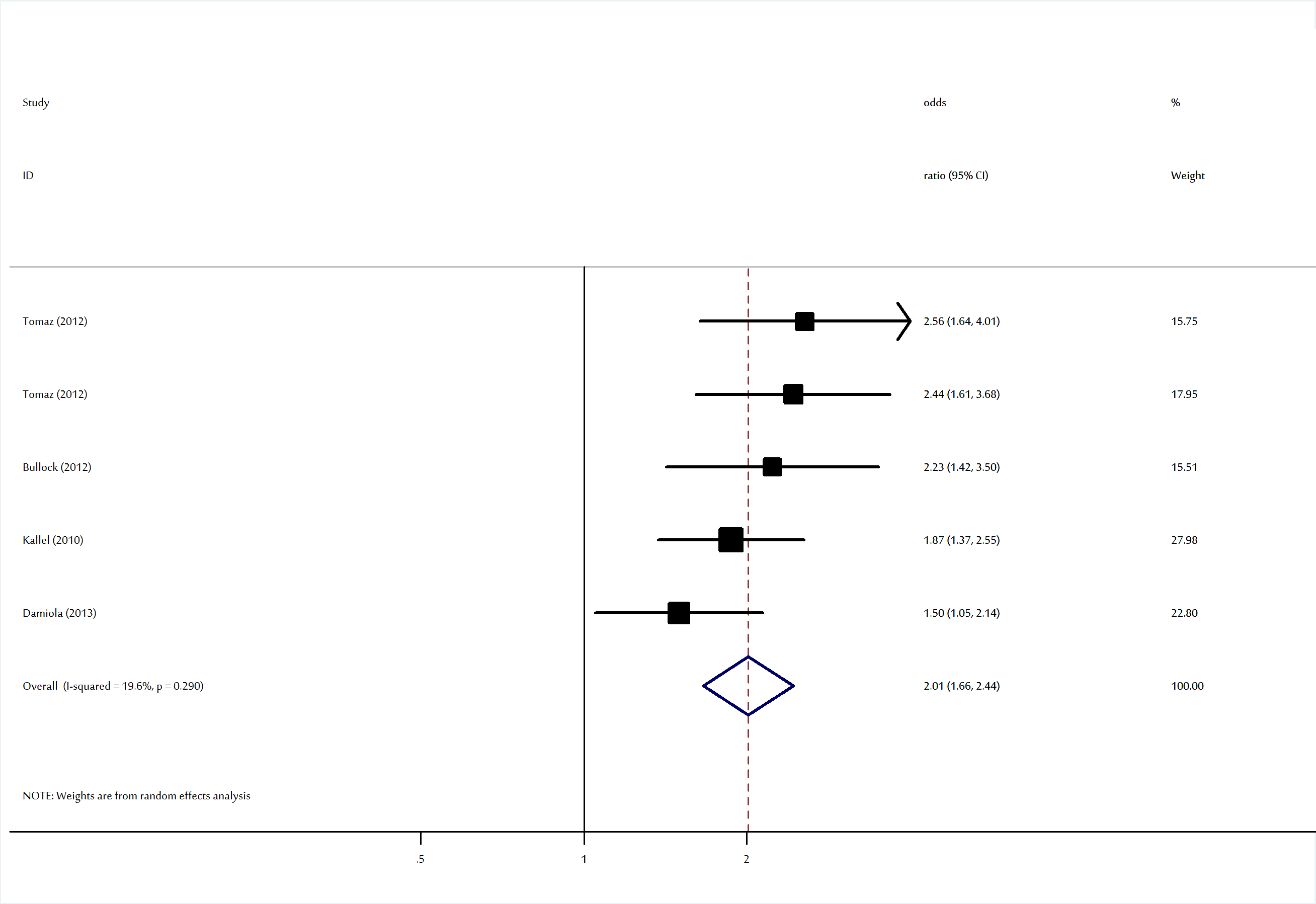

Supplement: Figure S4 — Forest plot for association of FOXE1 polyAla variant (71369530) and thyroid cancer risk. (TIF) [file pone.0087332.s004.tif]

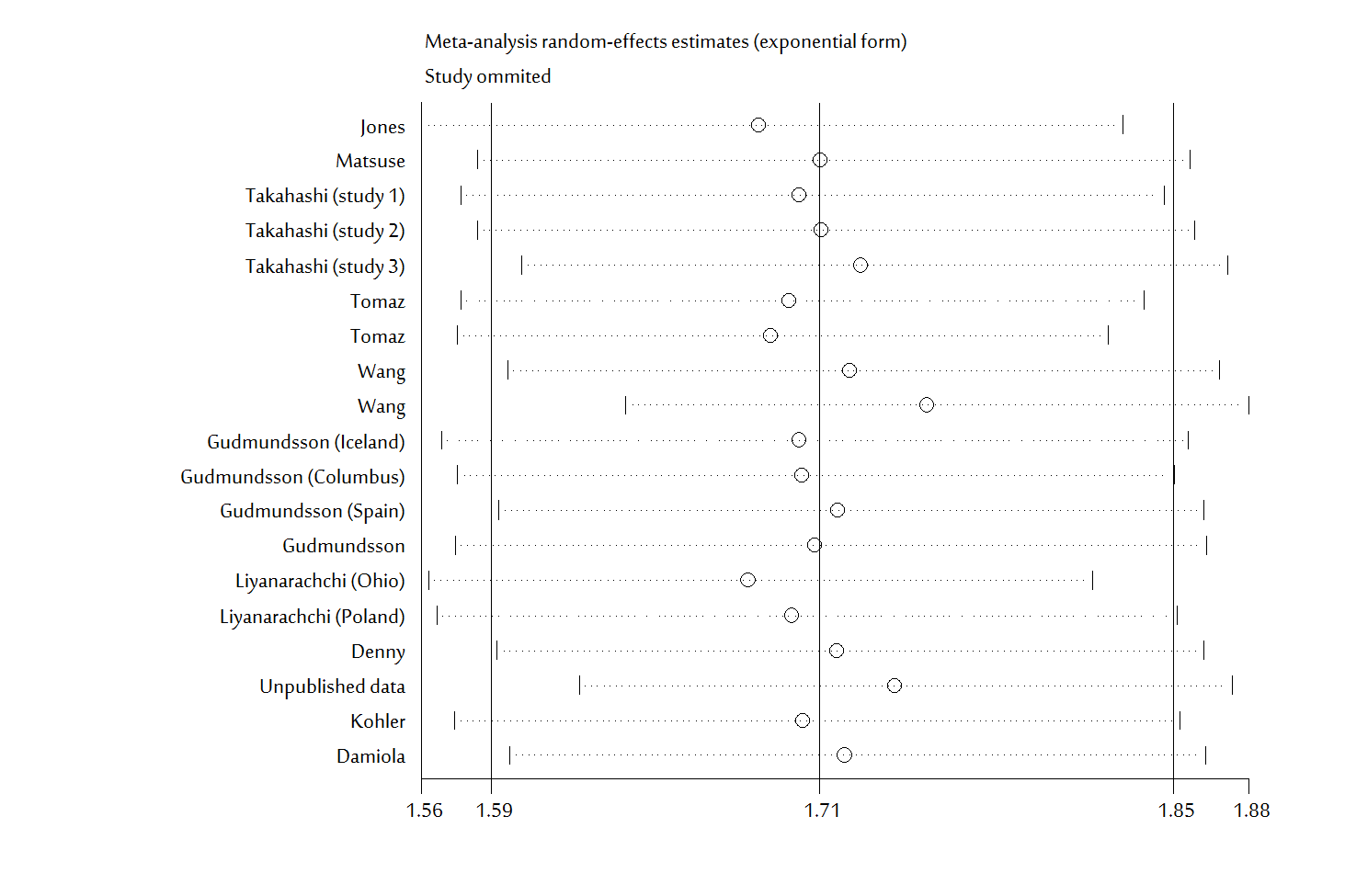

Supplement: Figure S5 — Result of sensitivity analyses for FOXE1 rs965513 polymorphism and thyroid cancer risk. (TIF) [file pone.0087332.s005.tif]

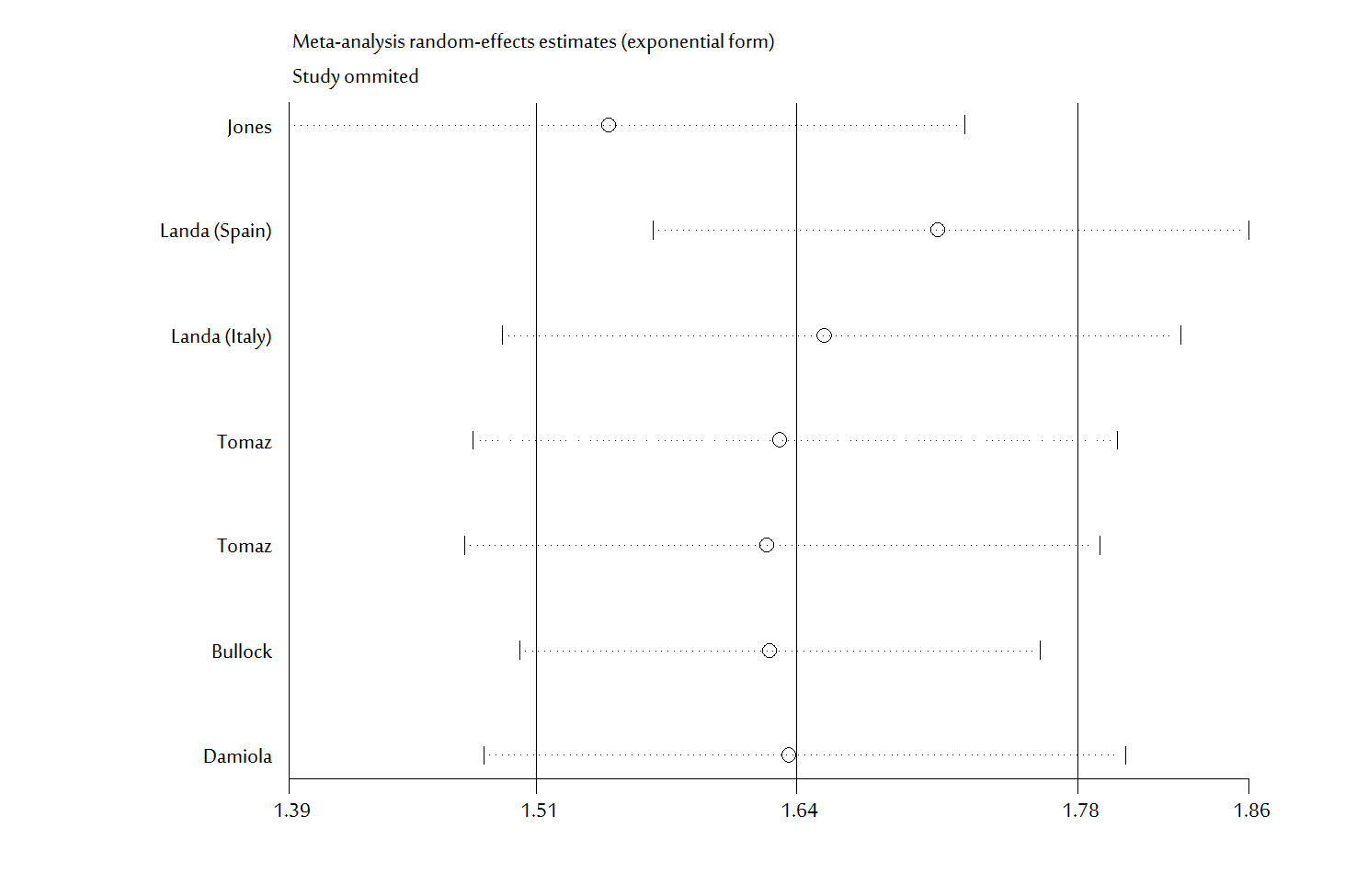

Supplement: Figure S6 — Result of sensitivity analyses for FOXE1 rs1867277 polymorphism and thyroid cancer risk. (TIF) [file pone.0087332.s006.tif]

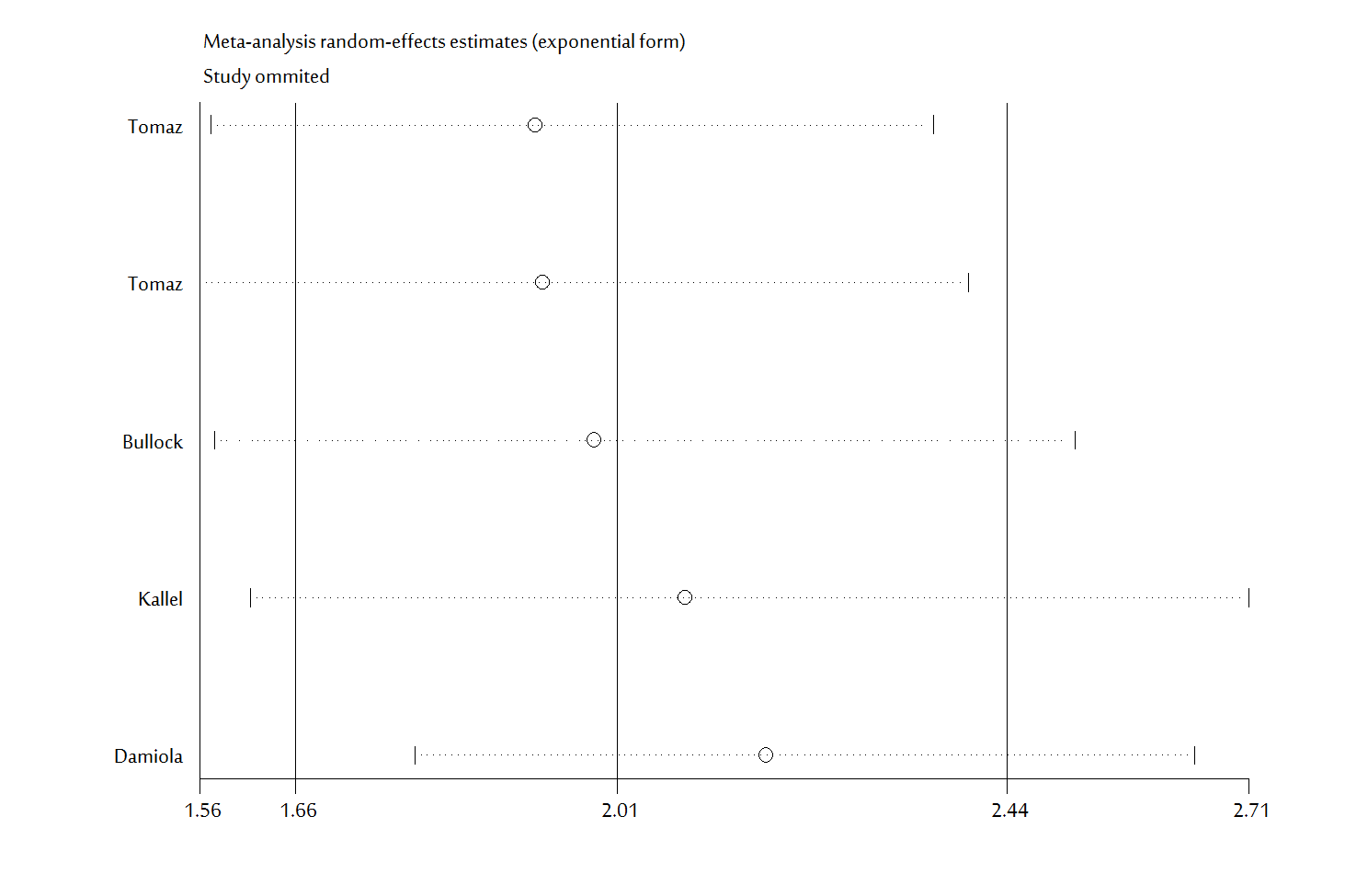

Supplement: Figure S7 — Result of sensitivity analyses for FOXE1 rs71369530 polymorphism and thyroid cancer risk. (TIF) [file pone.0087332.s007.tif]

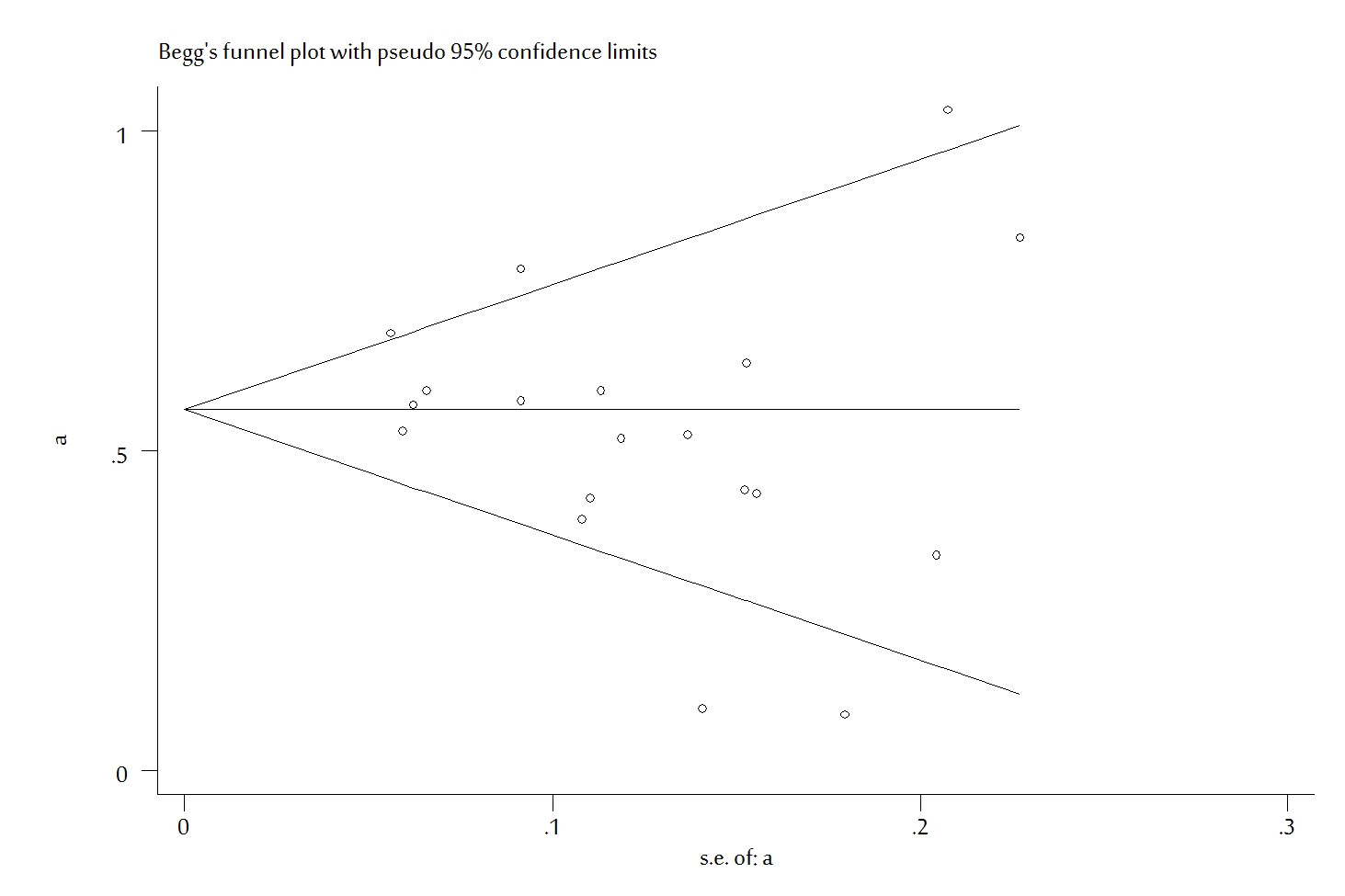

Supplement: Figure S8 — Begg's funnel plot for publication bias in studies on FOXE1 rs965513 polymorphism and thyroid cancer. (TIF) [file pone.0087332.s008.tif]

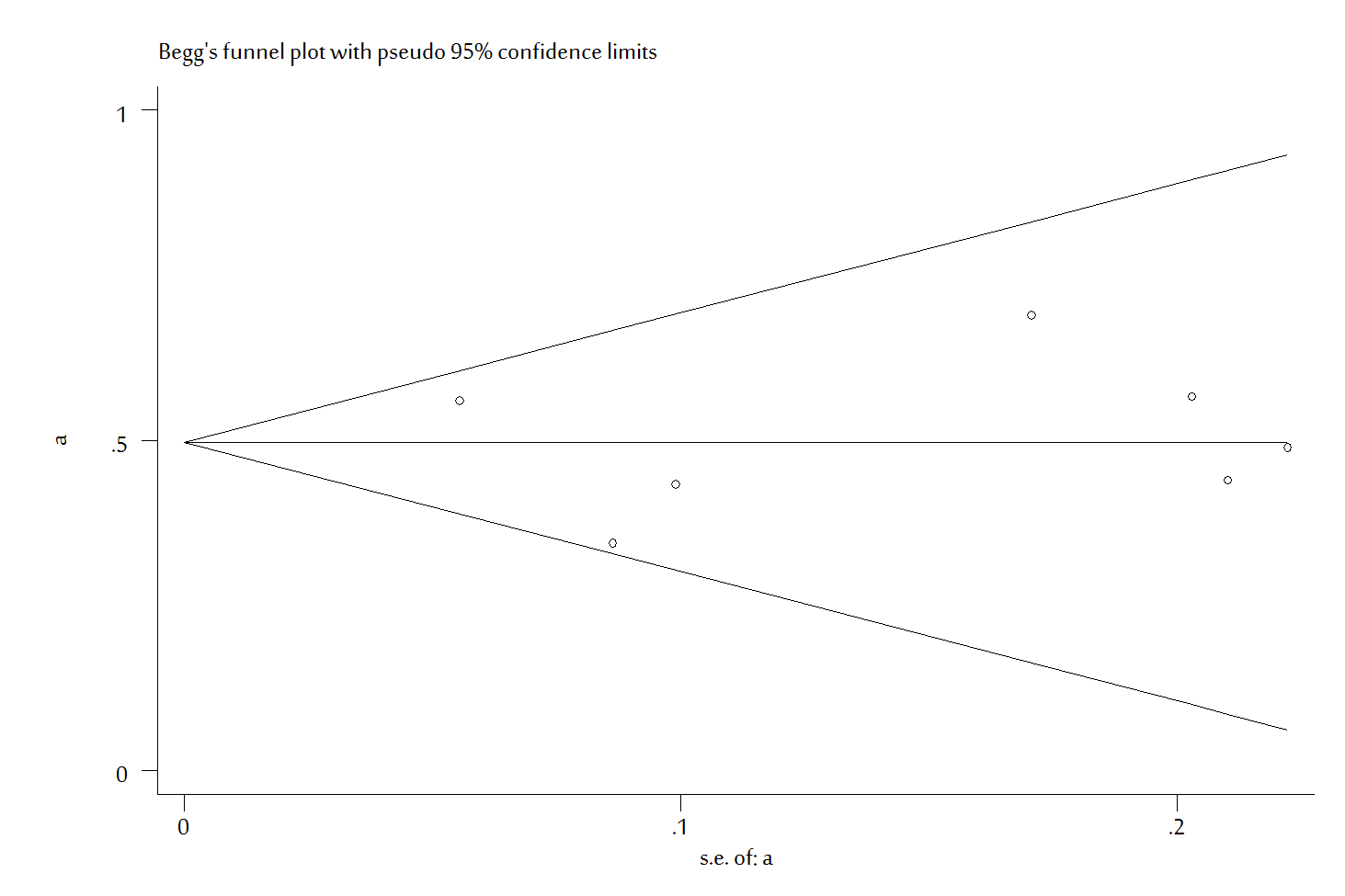

Supplement: Figure S9 — Begg's funnel plot for publication bias in studies on FOXE1 rs1867277 polymorphism and thyroid cancer. (TIF) [file pone.0087332.s009.tif]

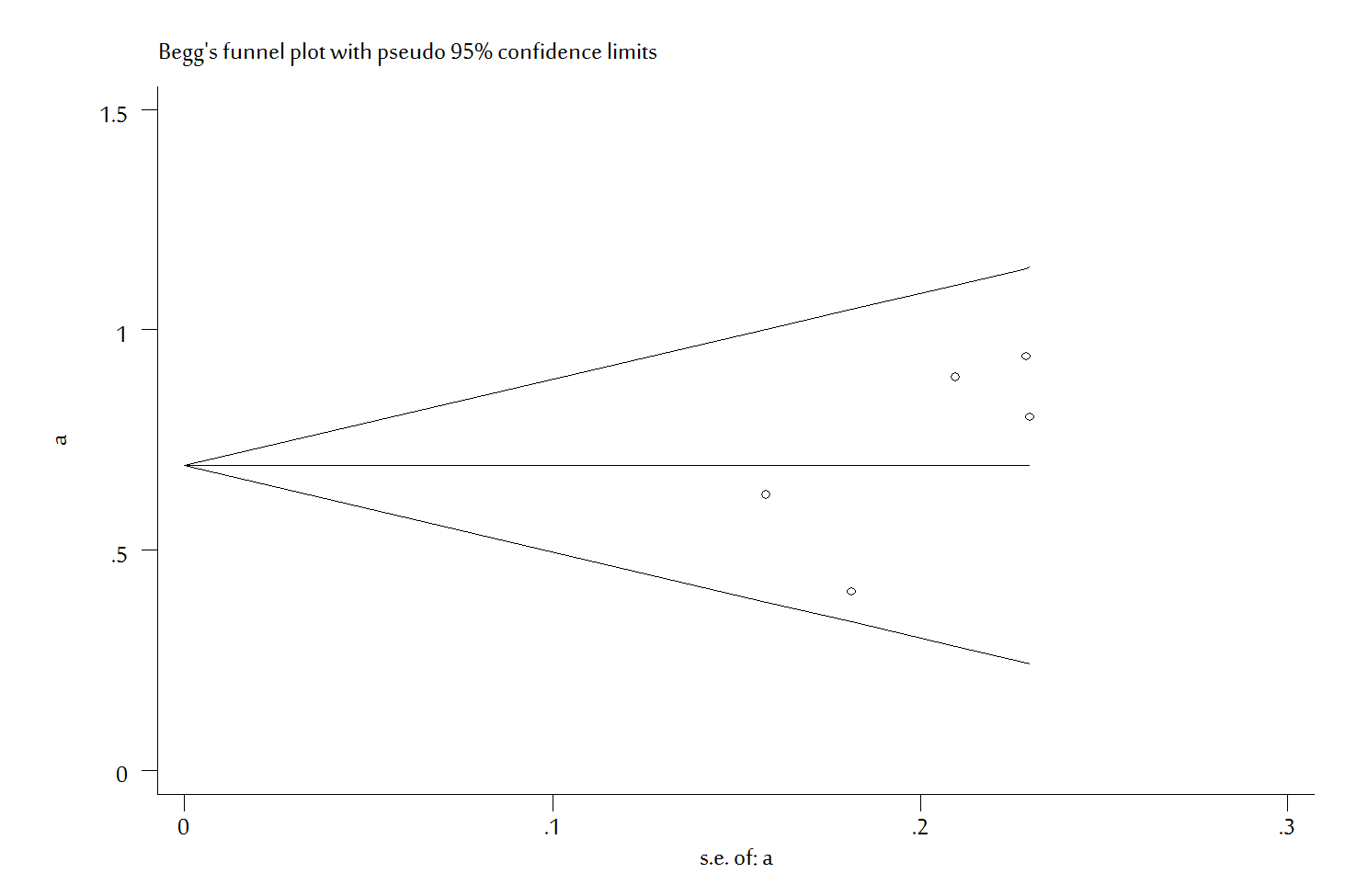

Supplement: Figure S10 — Begg's funnel plot for publication bias in studies on FOXE1 rs71369530 polymorphism and thyroid cancer. (TIF) [file pone.0087332.s010.tif]
